# Glycerol Monolaurate Inhibits Wild-Type African Swine Fever Virus Infection in Porcine Macrophages

**DOI:** 10.3390/pathogens12101193

**Published:** 2023-09-25

**Authors:** Joshua A. Jackman, Erik Arabyan, Hovakim Zakaryan, Charles C. Elrod

**Affiliations:** 1 School of Chemical Engineering and Translational Nanobioscience Research Center, Sungkyunkwan University, Suwon 16419, Republic of Korea; 2 Laboratory of Antiviral Drug Discovery, Institute of Molecular Biology of NAS, Yerevan 0014, Armenia; erik.arabyan.bio@gmail.com (E.A.); h_zakaryan@mb.sci.am (H.Z.); 3 Natural Biologics Inc., Newfield, NY 14867, USA; 4 Department of Animal Science, Cornell University, Ithaca, NY 14853, USA

**Keywords:** African swine fever virus, antiviral, mitigation, glycerol monolaurate, monoglyceride

## Abstract

Naturally abundant antimicrobial lipids, such as fatty acids and monoglycerides, that disrupt membrane-enveloped viruses are promising mitigants to inhibit African swine fever virus (ASFV). Among mitigant candidates in this class, glycerol monolaurate (GML) has demonstrated particularly high antiviral activity against laboratory-adapted ASFV strains. However, there is an outstanding need to further determine the effects of GML on wild-type ASFV strains, which can have different virulence levels and sensitivities to membrane-disrupting compounds as compared to laboratory-adapted strains. Herein, we investigated the antiviral effects of GML on a highly virulent strain of a wild-type ASFV isolate (Armenia/07) in an in vitro porcine macrophage model. GML treatment caused a concentration-dependent reduction in viral infectivity, and there was a sharp transition between inactive and active GML concentrations. Low GML concentrations had negligible effect on viral infectivity, whereas sufficiently high GML concentrations caused a >99% decrease in viral infectivity. The concentration onset of antiviral activity matched the critical micelle concentration (CMC) value of GML, reinforcing that GML micelles play a critical role in enabling anti-ASFV activity. These findings validate that GML can potently inhibit wild-type ASFV infection of porcine macrophages and support a biophysical explanation to guide antimicrobial lipid performance optimization for pathogen mitigation applications.

## 1. Introduction

The African swine fever virus (ASFV) is the causative agent of a highly lethal hemorrhagic disease with near 100% mortality in newly exposed pig populations and is a major agricultural biosecurity risk [1,2]. In recent years, ASFV outbreaks have significantly affected pig production worldwide, especially in parts of Asia where an epidemic occurred in 2018–2021, and have impacted global food and feed markets [3]. From a biosecurity perspective, ASFV is challenging to stop because there are no currently approved vaccines or therapeutics [4,5]. Hence, preventing ASFV transmission is critical and mainly relies on containment, sanitation, and surface disinfection [6,7,8]. Within this scope, there has been growing attention to the role that feed and drinking water can play as transmission vectors in contributing to ASFV disease spread [9] and in developing additive-based chemical mitigation strategies to inhibit ASFV in these matrices [10]. While formaldehyde is a widely used crosslinking additive to inhibit viral pathogens in such contexts, regulatory actions have banned its usage in certain jurisdictions, such as the European Union [Regulation (EU) 2018/183] (see also discussion in Ref. [11]), and there are ongoing efforts to develop new classes of regulatory acceptable mitigants with antiviral properties.

One promising target for antiviral mitigant development is the phospholipid membrane envelope that surrounds infectious ASFV particles [12]. Envelope targeting has two main advantages: (1) the lipid bilayer structure of the envelope is conserved across different virus strains so that one mitigant can broadly work against multiple virus strains in principle and potentially against different viruses; and (2) viruses cannot easily mutate to become resistant since the membrane components in the envelope are derived from host cell membranes and are not encoded in the viral genome [13,14]. Nevertheless, compared to other single-enveloped pig viruses, such as porcine epidemic diarrhea virus (PEDV) and porcine reproductive and respiratory syndrome virus (PRRSV), ASFV is a more rugged, double-enveloped virus belonging to the nucleocytoplasmic large DNA virus (NCLDV) family (Ref. [15]), and experimental testing on ASFV is thus needed to validate the antiviral efficacy of anti-ASFV mitigant candidates.

The need to test antiviral mitigants against ASFV while maintaining a high biosafety level has led to the development of the non-virulent ASFV BA71V strain, which is adapted to infect commonly used cell lines but neither infects porcine cells nor causes disease in pigs [16]. The ASFV BA71V strain has proven effective for testing membrane-disrupting antiviral mitigant candidates such as antimicrobial lipids (fatty acids and monoglycerides [17,18]) and rigid amphipathic fusion inhibitors (RAFIs) [19,20,21]. However, experimental data indicate that some membrane-disrupting mitigants can exhibit strain-specific antiviral activities. For example, certain RAFIs were shown to inhibit the ASFV BA71V strain by inhibiting virus-cell attachment, whereas the same compounds did not affect cellular attachment of the virulent, wild-type ASFV Armenia/07 strain isolate that infects porcine macrophages [19]. These findings underscore that the BA71V strain may be an effective screening tool (as are potentially other NCLDV surrogates under development as well [22,23]) to identify anti-ASFV mitigants, but further validation against virulent ASFV strains is needed to support translation.

Herein, we evaluated the antiviral properties of glycerol monolaurate (GML) against the virulent ASFV Armenia/07 strain in order to validate the potential antiviral efficacy of GML against circulating, wild-type ASFV strains. GML is a regulatory acceptable, food-grade monoglyceride that disrupts phospholipid membranes and has shown antiviral efficacy against a wide range of enveloped viruses, including in vivo treatment effects to ameliorate PEDV and Seneca Valley virus (SVV) infections in pigs [24,25]. Of note, GML has previously been shown to abrogate non-virulent ASFV BA71V strain infectivity in drinking water and had a higher level of antiviral activity than other tested medium-chain fatty acids [20]. Furthermore, GML blunted ASFV BA71V strain infectivity in feed while additionally causing conformational changes in viral surface proteins [20]. Building on these findings, our objective in the present study was to further explore the feasibility of GML to inhibit ASFV Armenia/07 infection in a porcine macrophage model, especially in terms of elucidating concentration-dependent effects that relate to the biophysical characteristics of GML as an antiviral mitigant.

## 2. Materials and Methods

### 2.1. Cell and Virus Preparations

The virulent ASFV Armenia/07 strain was used in all experiments, as previously described [26]. Viral titer quantification was conducted by the hemadsorption (HAD) assay, and titer levels are expressed in units of 50% hemadsorption doses (HADU_50_) per mL. In addition, primary porcine alveolar macrophages (PAMSs) were obtained and prepared following established protocols [27]. Prior to antiviral testing, the PAMs were maintained at 37 °C in Dulbecco’s modified Eagle’s medium that was supplemented with 10% fetal bovine serum, 2 mM L-glutamine, 100 IU/mL penicillin, and 100 μg/mL streptomycin. All kits and reagents were obtained from Sigma-Aldrich (Darmstadt, Germany) unless otherwise specified.

### 2.2. Cytotoxicity Assay

The effect of GML on PAM cell viability was investigated by the 3-(4,5-dimethylthiazol-2-yl)-2,5-diphenyltetrazolium bromide (MTT) assay. Cells in a 96-well cell culture plate (1 × 10^4^ cells per well) were treated with different GML concentrations (500, 250, 125, 63, or 31 µM in a two-fold dilution series). Treated cells were incubated for up to 72 h at 37 °C in a 5% CO_2_ environment. After incubation, the medium was removed and MTT solution was added. The microplates were incubated at 37 °C for 3 h after adding MTT solution, followed by purple formazan extraction by MTT solvent. The colorimetric measurements were performed on a microplate reader at 570 nm. The percentage of viable cells was calculated at each GML concentration as [(ODT/ODC) × 100%], whereby ODT and ODC correspond to the absorbance (optical density) of treated and control cells, respectively.

### 2.3. Antiviral Assay

Suspensions of ASFV Armenia/07 were prepared at a multiplicity of infection (MOI) of 0.5 or 1 HADU_50_ per well and were treated with different GML concentrations (250, 125, 63, or 31 µM in a two-fold dilution series) for one hour at room temperature along with negative control (virus-only without GML). GML is understood to quickly disrupt phospholipid membranes (<15 min), and the time period was selected to ensure sufficient incubation time in line with past reports [20].

The treated virus samples were then added to infect PAM cells seeded at 2 × 10^5^ cells per well in a 24-well plate and were cultured for 48 or 72 h. Cell culture supernatants were collected at 24, 48, and/or 72 h post-infection as appropriate, and viral titer in the supernatant was quantified by HAD assay upon porcine erythrocyte addition [27]. ASFV presence was quantified by counting the formation of erythrocyte rosettes around infected macrophages and was expressed in HADU_50_/mL units accordingly.

### 2.4. Statistical Analysis 

All statistical tests were performed using the GraphPad Prism 8 software package (San Diego, CA, USA). One-way analysis of variance (ANOVA) with Dunnett’s multiple comparisons test (versus virus-only control) was used. Statistical significance was computed in terms of multiplicity-adjusted *p* values, and *p* < 0.05 indicates the cutoff for statistical significance. 

## 3. Results and Discussion

We selected the ASFV Armenia/07 strain as the model virulent strain for assessing the antiviral activity of GML, which is a saturated monoglyceride that is an esterified adduct of 12-carbon-long lauric acid and glycerol. GML was originally identified to possess potent antimicrobial activity for inhibiting bacteria compared to other medium-chain fatty acids and monoglycerides, and more recently it has been explored for inhibiting enveloped viruses as well [28,29]. From biophysical experiments, it is understood that GML mainly disrupts phospholipid membranes at concentrations above its critical micelle concentration (CMC) (Ref. [30]), and membrane disruption is understood to be the basis for its antiviral activity against enveloped viruses [31]. Our selection of the ASFV Armenia/07 strain in particular was further motivated by two main factors: (1) this strain isolate is highly virulent in pigs and similar to the wild-type ASFV Georgia/07 strain that initially caused an outbreak in the Caucasus region [32,33]; and (2) the strain has been used previously to test other membrane-disrupting antiviral molecules such as RAFIs [19]. Accordingly, ASFV virus suspensions (MOI: 0.5 HADU_50_ per well) in aqueous solution were mixed with different GML concentrations for a treatment period, followed by adding the virus–GML mixtures to infect PAM cells. The infection process was allowed to continue, and cell culture media supernatants were collected at 48 and 72 h post-infection in order to measure the extent of infectious virus replication. The GML concentration range was chosen based on the range of antiviral potency observed in past testing with the ASF BA71V strain (31–250 μM) [20] and also bridges the CMC range of GML (~60 μM) [34].

**Figure 1:**
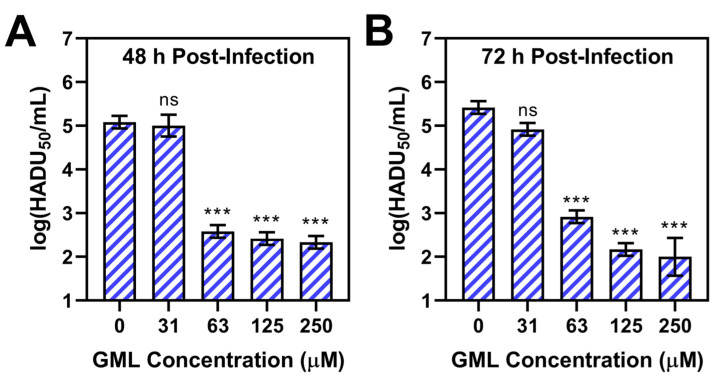
Antiviral activity of GML to inhibit Armenia/07 ASFV infection of porcine macrophages in vitro. The virus suspension was treated with different concentrations of GML (31–250 μM) prior to PAM cell infection. The 0 μM GML data point corresponds to the virus-only control. Viral titers of cell culture supernatants were measured (**A**) 48 h or (**B**) 72 h post-infection by hemadsorption assay. Data are reported in units of log 50% hemadsorption doses (HADU_50_) per mL and presented as mean ± standard deviation from three independent experiments (*n* = 3 per group). The markers *** and ns indicate *p* < 0.001 and *p* > 0.05, respectively, as compared to the virus-only control.

At 48 h post-infection, the virus-only control titer was 5.1 ± 0.1 log(HADU_50_/mL), and the titer of the virus sample treated with 31 μM GML was similar at around 5.0 ± 0.3 log(HADU_50_/mL) (Figure 1A). In marked contrast, virus samples treated with 63, 125, or 250 μM GML had significantly reduced titers around 2.6 ± 0.1, 2.4 ± 0.1, and 2.3 ± 0.1 log(HADU_50_/mL), respectively. Thus, GML had a negligible antiviral activity until reaching a critical concentration of 63 μM, which is around the CMC value of GML. This concentration-dependent finding agrees well with past ASFV BA71V testing results [20] and supports that GML micelles are the principal unit involved in disrupting ASFV particles.

Similar test results were also recorded at 72 h post-infection (Figure 1B). In this case, the virus-only control titer was 5.4 ± 0.1 log(HADU_50_/mL), whereas the titer of the virus sample treated with 31 μM GML was 4.9 ± 0.1 log(HADU_50_/mL). While the average titer difference was 0.5 log units, the difference was not statistically significant. By contrast, virus samples treated with 63, 125, or 250 μM GML had significantly reduced titers around 2.9 ± 0.1, 2.2 ± 0.1, and 2.0 ± 0.4 log(HADU_50_/mL), respectively. 

We also performed similar experiments at a higher MOI (1 HADU_50_ per well) and observed a similar trend in the concentration-dependent antiviral effects of GML (Figure S1). At 24 h post-infection, the virus-only control titer was ~5.5 log(HADU_50_/mL), and a similar titer was also recorded for the virus sample treated with 31 μM GML. Conversely, virus samples treated with 63, 125, or 250 μM GML tended to have reduced titers of around ~4.7, ~4.2, and ~3.7 log(HADU_50_/mL), respectively. At 48 h infection, a similar trend was again observed. For the virus-only control and virus sample treated with 31 μM GML, the titers were around ~6.3 log(HADU_50_/mL), whereas virus samples treated with 63, 125, or 250 μM GML tended to have decreased titers of around ~4.2, ~4.4, and ~3.9 log(HADU_50_/mL), respectively. Taken together, these data reinforce that GML was only active at and above 63 μM, whereas a two-fold reduction in the GML concentration led to an insignificant antiviral effect.

**Figure 2:**
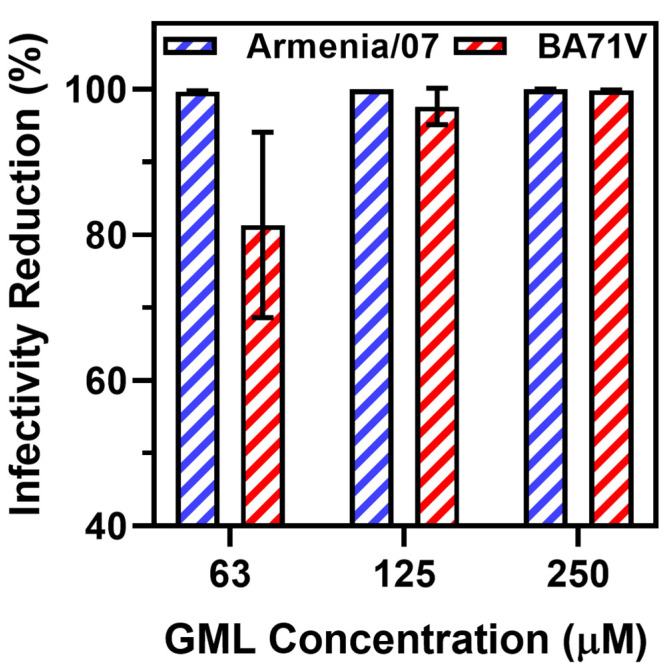
Quantitative analysis of GML inhibitory effect in vitro on virulent (Armenia/07) and non-virulent (BA71V) ASFV strains. The concentration-dependent inhibitory effects of GML are expressed in terms of the degree of infectivity reduction relative to virus-only controls. The Armenia/07 strain titers were measured in terms of HADU_50_/mL units, and the BA71V strain titers were measured in terms of 50% tissue culture infective dose (TCID_50_) per mL units (quantitative analysis was done on raw data from Figure S1 of Ref. [20]). Data are presented as mean ± standard deviation from three independent experiments (*n* = 3 per group).

In addition to changes in viral titer, we also plotted the percentages of viral infectivity relative to the virus-only control (Figure 2). This quantification approach allowed us to obtain unitless indicators of viral infectivity reduction in order to plot data obtained with the virulent ASFV Armenia/07 strain here and data obtained with the non-virulent ASFV BA71V strain in a past study [20]. While the absolute viral titers cannot be compared directly because different methods were used depending on the virus strain and cell type, we focused on calculating the percentage change in viral infectivity relative to the appropriate untreated control. We first analyzed the antiviral data collected with the ASFV Armenia/07 strain at 72 h post-infection and focused on ≥63 μM GML treatment conditions, within which range statistically significant antiviral effects were observed. Within this range, 63, 125, and 250 μM GML treatments caused significant drops in viral infectivity of around 99.7% ± 0.10%, 99.9% ± 0.02%, and 99.9% ± 0.06%, respectively.

These data were plotted alongside previously obtained antiviral data that evaluated the effects of GML treatment on ASFV BA71V strain infectivity in a permissive Vero cell model by using a cytopathic effect assay. In those experiments, 31 μM GML treatment had a negligible effect on viral infectivity relative to the virus-only control, whereas 63, 125, and 250 μM GML treatments caused significant drops in viral infectivity of around 81.3% ± 12.7%, 97.6% ± 2.5%, and 99.8% ± 0.06%, respectively. 

Collectively, the data support that GML only exhibited antiviral activity against both ASFV strains at and above 63 μM GML concentration, which supports that GML micelles are the main membrane-disrupting species to inhibit ASFV. This finding fits with past biophysical studies that showed that GML only disrupts phospholipid bilayers at and above its CMC, whereas GML monomers had a negligible effect [30,35]. Furthermore, these findings support that GML exhibits similar levels of antiviral potency (i.e., the lowest GML concentration at which antiviral activity occurs to a significant extent) against the virulent ASFV Armenia/07 and the non-virulent ASFV BA71V strains, and this concentration dependency supports that GML micelles mainly contribute to antiviral activity against both virus strains. This latter conclusion is reinforced by two findings: (1) the concentration onset at which antiviral activity occurs; and (2) the sharp transition between inactive and active GML concentrations.

While acknowledging that the antiviral tests involving ASFV Armenia/07 and ASFV BA71V strains were conducted on different cell lines and with different assays and that a direct comparison between antiviral tests on the two strains should not be made for this reason, we may also briefly comment on the different degrees of viral inactivation that GML exhibited against each strain. If we treat a 3 log drop in viral infectivity (99.9%) reduction as a performance cutoff, then the data support that treatment of ASFV Armenia/07 with ≥125 μM GML meets this performance threshold (≥63 μM GML for a 99% threshold). In addition, treatment with 250 μM GML met the 99% performance threshold for inhibiting the ASFV BA71V strain. Across the two independent sets of experimental data, it should be emphasized that the effect of GML on reducing ASFV infectivity in both strains is quite high. For SVV, it has been reported that GML treatment yielded a maximum reduction of only ~80% in in vitro experiments, yet still resulted in good in vivo performance in a porcine model in terms of reducing clinical symptoms, viral loads, and organ damage as well as promoting positive inflammatory responses [25]. GML exhibited a similar inhibitory effect on PRRSV, with around 80% reduction in viral infectivity in vitro [36].

**Figure 3:**
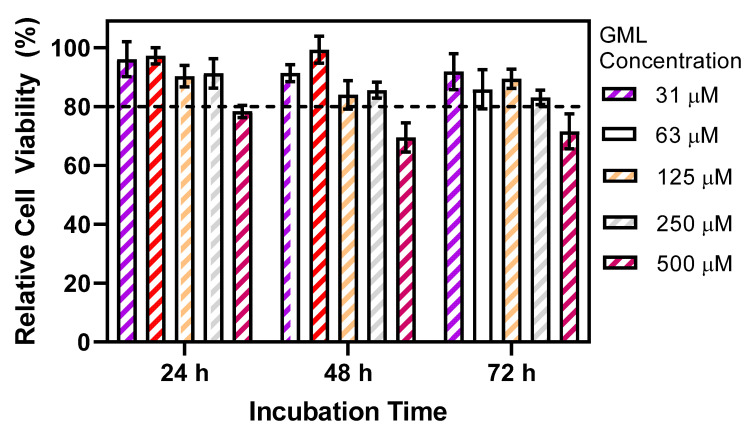
Effect of GML concentration on PAM cell viability. Different GML concentrations were incubated with PAM cells for 24, 48, or 72 h prior to MTT analysis. Data are expressed in terms of relative cell viability compared to negative control (no GML) and are presented as mean ± standard deviation from three independent experiments (*n* = 3 per group). The horizontal dashed line corresponds to a 20% cutoff drop in relative cell viability.

In addition to antiviral tests, we also investigated the concentration-dependent effects of GML on PAM cell viability (Figure 3). While the envisioned antiviral applications of GML are mainly aimed at ex vivo mitigation (e.g., in drinking water), these cell cytotoxicity experiments were directed at confirming sufficient PAM cell viability in the presence of GML within the tested concentration range and also at distinguishing between the effects of GML on abiotic membranes, such as those of enveloped viruses that lack reparative capacity as described above, vs. on biotic membranes, such as those of mammalian cells that have reparative capacity (as previously discussed in the context of other classes of membrane-disrupting antivirals, such as RAFIs [13,37]). 

While ASFV infectivity decreased significantly when treated with GML at and above its CMC, PAM cell viability remained similarly high in this GML concentration range, and appreciable cytotoxicity (>20% drop in viability) only occurred at higher GML concentrations outside the antiviral test range. After 24 h incubation, cell viability upon treatment with 31–250 µM GML was >90% and only dropped to <80% viability at 500 µM GML. This trend is generally consistent with past reports describing how the 50% cell cytotoxicity values of GML against human lung fibroblasts and skin keratinocytes did not match the CMC and were in the range of ~300 µM (Ref. [38]), supporting that the effects of GML on PAM cell viability are not CMC-dependent. Similar trends in cell viability (>80% viability up to 250 µM GML) were observed after 48 and 72 h post-incubation. These findings support that the CMC-dependent antiviral activity of GML to inhibit ASFV is related to GML-micelle-induced irreparable membrane disruption of the viral envelope, while further suggesting that GML micelles have less deleterious effects on PAM cells with reparative membrane capacity.

## 4. Conclusions

In summary, the findings in this study support that GML can inhibit wild-type ASFV strains and that the mechanism of antiviral activity depends on GML micelle formation. While there has been recent discussion about how micelle formation may not be an absolute prerequisite for the antiviral activity of surfactant-like molecules against enveloped viruses in all cases and modest antiviral activity can sometimes be observed at slightly lower concentrations below CMC (as described in the context of searching for Triton X-100 replacements [39]), the results obtained in the present study indicate that micelle formation is a key contributing factor to the antiviral activity of GML, which is also consistent with the membrane biophysics literature. From a translational perspective, these findings further emphasize the importance of organizing GML into supramolecular assemblies, whereby the effects of GML in self-assembled nanostructures are greater than the effects of GML monomers. Above CMC, GML micelles are spontaneously formed self-assembled nanostructures; however, they can collapse upon dilution, e.g., upon administration into an animal or injection into a drinking water line. Thus, incorporating GML into dilution-stable nanostructures, such as solid lipid nanoparticles [40], may represent a future opportunity to harness its antiviral activity in additional types of supramolecular assemblies beyond micelles and may support the use of GML in pathogen-mitigation applications across feed and drinking water matrices.

## Data Availability

The data presented in this study are available upon reasonable request from the corresponding authors.

## Supplementary Materials

The following supporting information can be downloaded at: https://www.mdpi.com/article/10.3390/pathogens12101193/s1, Figure S1: Antiviral activity of GML to inhibit Armenia/07 ASFV infection of porcine macrophages in vitro at higher multiplicity of infection.

## References

[1] Galindo I., Alonso C. (2017). African swine fever virus: A review. Viruses.

[2] Gaudreault N.N., Madden D.W., Wilson W.C., Trujillo J.D., Richt J.A. (2020). African swine fever virus: An emerging DNA arbovirus. Front. Vet. Sci..

[3] You S., Liu T., Zhang M., Zhao X., Dong Y., Wu B., Wang Y., Li J., Wei X., Shi B. (2021). African swine fever outbreaks in China led to gross domestic product and economic losses. Nat. Food.

[4] Arabyan E., Kotsynyan A., Hakobyan A., Zakaryan H. (2019). Antiviral agents against African swine fever virus. Virus Res..

[5] Penrith M.L., Kivaria F.M. (2022). One hundred years of African swine fever in Africa: Where have we been, where are we now, where are we going?. Transbound. Emerg. Dis..

[6] Barongo M.B., Bishop R.P., Fèvre E.M., Knobel D.L., Ssematimba A. (2016). A mathematical model that simulates control options for African swine fever virus (ASFV). PLoS ONE.

[7] Mutua F., Dione M. (2021). The context of application of biosecurity for control of African swine fever in smallholder pig systems: Current gaps and recommendations. Front. Vet. Sci..

[8] Beato M.S., D’Errico F., Iscaro C., Petrini S., Giammarioli M., Feliziani F. (2022). Disinfectants against African swine fever: An updated review. Viruses.

[9] Niederwerder M.C., Stoian A.M., Rowland R.R., Dritz S.S., Petrovan V., Constance L.A., Gebhardt J.T., Olcha M., Jones C.K., Woodworth J.C. (2019). Infectious dose of African swine fever virus when consumed naturally in liquid or feed. Emerg. Infect. Dis..

[10] Phillips F.C., Rubach J.K., Poss M.J., Anam S., Goyal S.M., Dee S.A. (2022). Monoglyceride reduces viability of porcine epidemic diarrhoea virus in feed and prevents disease transmission to post-weaned piglets. Transbound. Emerg. Dis..

[11] Śmiechowska M., Newerli-Guz J., Skotnicka M. (2021). Spices and seasoning mixes in European Union—Innovations and ensuring safety. Foods.

[12] Wang N., Zhao D., Wang J., Zhang Y., Wang M., Gao Y., Li F., Wang J., Bu Z., Rao Z. (2019). Architecture of African swine fever virus and implications for viral assembly. Science.

[13] Vigant F., Santos N.C., Lee B. (2015). Broad-spectrum antivirals against viral fusion. Nat. Rev. Microbiol..

[14] Yoon B.K., Jeon W.-Y., Sut T.N., Cho N.-J., Jackman J.A. (2021). Stopping membrane-enveloped viruses with nanotechnology strategies: Toward antiviral drug development and pandemic preparedness. ACS Nano.

[15] Hernáez B., Guerra M., Salas M.L., Andrés G. (2016). African swine fever virus undergoes outer envelope disruption, capsid disassembly and inner envelope fusion before core release from multivesicular endosomes. PLoS Pathog..

[16] Rodríguez J.M., Moreno L.T., Alejo A., Lacasta A., Rodríguez F., Salas M.L. (2015). Genome sequence of African swine fever virus BA71, the virulent parental strain of the nonpathogenic and tissue-culture adapted BA71V. PLoS ONE.

[17] Jackman J.A., Boyd R.D., Elrod C.C. (2020). Medium-chain fatty acids and monoglycerides as feed additives for pig production: Towards gut health improvement and feed pathogen mitigation. J. Anim. Sci. Biotechnol..

[18] Jackman J.A., Lavergne T.A., Elrod C.C. (2022). Antimicrobial monoglycerides for swine and poultry applications. Front. Anim. Sci..

[19] Hakobyan A., Galindo I., Nañez A., Arabyan E., Karalyan Z., Chistov A.A., Streshnev P.P., Korshun V.A., Alonso C., Zakaryan H. (2018). Rigid amphipathic fusion inhibitors demonstrate antiviral activity against African swine fever virus. J. Gen. Virol..

[20] Jackman J.A., Hakobyan A., Zakaryan H., Elrod C.C. (2020). Inhibition of African swine fever virus in liquid and feed by medium-chain fatty acids and glycerol monolaurate. J. Anim. Sci. Biotechnol..

[21] Niederwerder M.C., Dee S., Diel D.G., Stoian A.M.M., Constance L.A., Olcha M., Petrovan V., Patterson G., Cino-Ozuna A.G., Rowland R.R.R. (2021). Mitigating the risk of African swine fever virus in feed with anti-viral chemical additives. Transbound. Emerg. Dis..

[22] Palowski A. (2021). The Use of a Risk-Free In Situ Non-Animal (RISNA) Surrogate Assay for Evaluating Inactivation Strategies of African Swine Fever Virus in Feed Ingredients in Real-World Demonstrations. Master’s Thesis.

[23] Palowski A., Balestreri C., Urriola P.E., van de Ligt J.L.G., Sampedro F., Dee S., Shah A., Yancy H.F., Shurson G.C., Schroeder D.C. (2022). Survival of a surrogate African swine fever virus-like algal virus in feed matrices using a 23-day commercial United States truck transport model. Front. Microbiol..

[24] Liu Z., Zhu L., Zhao X., Liu J., Cheng H., Zhang L., Tang H., Sun X., Hu Y., Xu Z. (2022). Effects of oral of administration of monoglycide laurate on virus load and inflammation in PEDV infected porcine. Front. Vet. Sci..

[25] Su B., Wang Y., Jian S., Tang H., Deng H., Zhu L., Zhao X., Liu J., Cheng H., Zhang L. (2023). In vitro and in vivo antiviral activity of monolaurin against Seneca Valley virus. Front. Vet. Sci..

[26] Arabyan E., Hakobyan A., Hakobyan T., Grigoryan R., Izmailyan R., Avetisyan A., Karalyan Z., Jackman J.A., Ferreira F., Elrod C.C. (2021). Flavonoid library screening reveals kaempferol as a potential antiviral agent against African swine fever virus. Front. Microbiol..

[27] Carrascosa A.L., Bustos M.J., de Leon P. (2011). Methods for growing and titrating African swine fever virus: Field and laboratory samples. Curr. Protoc. Cell Biol..

[28] Kabara J.J., Swieczkowski D.M., Conley A.J., Truant J.P. (1972). Fatty acids and derivatives as antimicrobial agents. Antimicrob. Agents Chemother..

[29] Welch J.L., Xiang J., Okeoma C.M., Schlievert P.M., Stapleton J.T. (2020). Glycerol monolaurate, an analogue to a factor secreted by lactobacillus, is virucidal against enveloped viruses, including HIV-1. mBio.

[30] Yoon B.K., Jackman J.A., Kim M.C., Cho N.-J. (2015). Spectrum of membrane morphological responses to antibacterial fatty acids and related surfactants. Langmuir.

[31] Hierholzer J.C., Kabara J.J. (1982). In vitro effects of monolaurin compounds on enveloped RNA and DNA viruses. J. Food Saf..

[32] Rowlands R.J., Michaud V., Heath L., Hutchings G., Oura C., Vosloo W., Dwarka R., Onashvili T., Albina E., Dixon L.K. (2008). African swine fever virus isolate, Georgia, 2007. Emerg. Infect. Dis..

[33] Sunwoo S.-Y., Pérez-Núñez D., Morozov I., Sánchez E.G., Gaudreault N.N., Trujillo J.D., Mur L., Nogal M., Madden D., Urbaniak K. (2019). DNA-protein vaccination strategy does not protect from challenge with African swine fever virus Armenia 2007 strain. Vaccines.

[34] Yoon B.K., Park S., Ma G.J., Kolahdouzan K., Zhdanov V.P., Jackman J.A., Cho N.-J. (2020). Competing interactions of fatty acids and monoglycerides trigger synergistic phospholipid membrane remodeling. J. Phys. Chem. Lett..

[35] Tan S.W., Jeon W.-Y., Yoon B.K., Jackman J.A. (2022). Mechanistic evaluation of antimicrobial lipid interactions with tethered lipid bilayers by electrochemical impedance spectroscopy. Sensors.

[36] Yang L., Wen J., Zhang Y., Liu Z., Luo Z., Xu L., Lai S., Tang H., Sun X., Hu Y. (2022). The antiviral activity of caprylic monoglyceride against porcine reproductive and respiratory syndrome virus in vitro and in vivo. Molecules.

[37] Vigant F., Hollmann A., Lee J., Santos N.C., Jung M.E., Lee B. (2014). The rigid amphipathic fusion inhibitor dUY11 acts through photosensitization of viruses. J. Virol..

[38] Yoon B.K., Jackman J.A., Park S., Mokrzecka N., Cho N.-J. (2019). Characterizing the membrane-disruptive behavior of dodecylglycerol using supported lipid bilayers. Langmuir.

[39] Farcet J.-B., Karbiener M., Zelger L., Kindermann J., Kreil T.R. (2023). Detergent-mediated virus inactivation in biotechnological matrices: More than just CMC. Int. J. Mol. Sci..

[40] Tan J.Y.B., Yoon B.K., Cho N.-J., Lovrić J., Jug M., Jackman J.A. (2021). Lipid nanoparticle technology for delivering biologically active fatty acids and monoglycerides. Int. J. Mol. Sci..

