# Supplementary material for: Glycerol Monolaurate Inhibits Wild-Type African Swine Fever Virus Infection in Porcine Macrophages
Source: Pathogens. 2023 Sep 25;12(10):1193. doi: 10.3390/pathogens12101193 (PMC10610281; doi:10.3390/pathogens12101193)
Supplement: Supplementary file 1 [file pathogens-12-01193-s001.zip › pathogens-2569070-supplementary.pdf]

Supplementary Materials

Glycerol Monolaurate Inhibits Wild-Type African Swine Fever Virus Infection in Porcine Macrophages

Joshua A. Jackman[1,*], Erik Arabyan[2], Hovakim Zakaryan[2] and Charles C. Elrod[3,4,*]

[1] School of Chemical Engineering and Translational Nanobioscience Research Center, Sungkyunkwan University, Suwon, 16419, Republic of Korea
[2] Laboratory of Antiviral Drug Discovery, Institute of Molecular Biology of NAS, Yerevan, Armenia; erik.arabyan.bio@gmail.com (E.A.); h_zakaryan@mb.sci.am (H.Z.)
[3] Natural Biologics Inc., Newfield, NY, 14867, USA
[4] Department of Animal Science, Cornell University, Ithaca, NY, 14853, USA
***** Correspondence: jjackman@skku.edu (J.A.J.) and cce1@cornell.edu (C.C.E.)

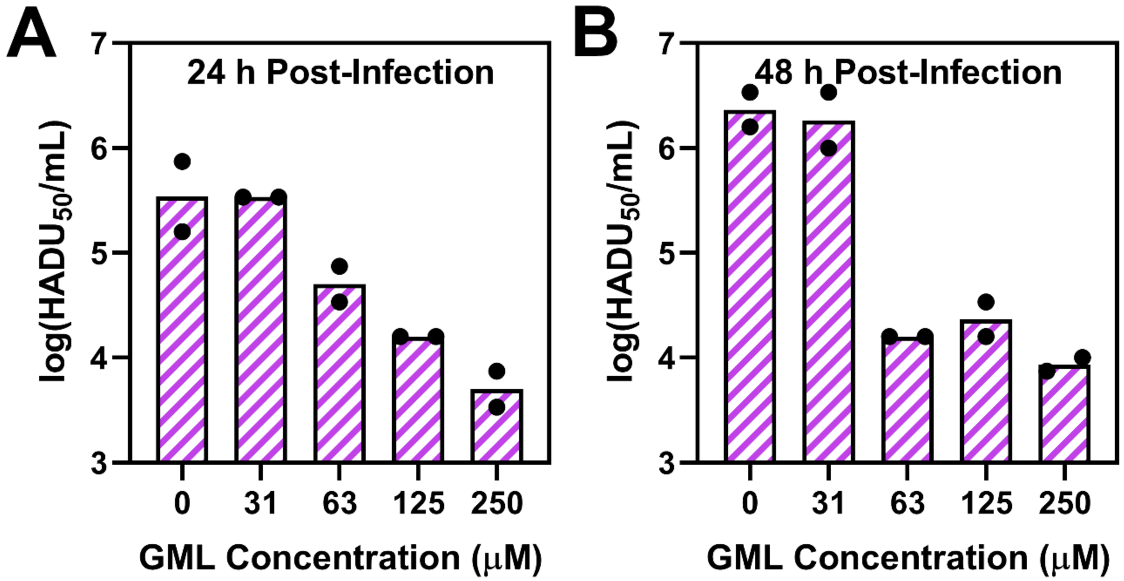

Figure S1. Antiviral activity of GML to inhibit Armenia/07 ASFV infection of porcine macrophages *in vitro* at higher multiplicity of infection. The virus suspension (MOI: 1 $HADU_{50}$ per well) was treated with different concentrations of GML (31-250 μM) prior to PAM cell infection. The 0 μM GML data point corresponds to the virus-only control. Viral titers of cell culture supernatants were measured **(A)** 24 h or **(B)** 48 h post-infection by hemadsorption assay. Data are reported in units of log 50% hemadsorption doses ($HADU_{50}$) per mL and presented as mean from two independent experiments (n = 2 per group). The dots correspond to individual data points.
